# Management and Treatment of Carotid Stenosis: Overview of Therapeutic Possibilities and Comparison Between Interventional Radiology, Surgery and Hybrid Procedure

**DOI:** 10.3390/diagnostics15131679

**Published:** 2025-07-01

**Authors:** Lorenzo Aliotta, Livio Maria Gavazzi, Pierantonio Malfa, Pietro Valerio Foti, Stefano Palmucci, Maria Chiara Lo Greco, Corrado Spatola, Corrado Inì, Francesco Tiralongo, Davide Castiglione, Rita Bella, Gianluca Galvano, Giuseppe Lanza, Silvia Gigli, Antonio Basile, Vito Cantisani, Emanuele David

**Affiliations:** 1 Department of Medical Surgical Sciences and Advanced Technologies “GF Ingrassia”, University Hospital Policlinic “G. Rodolico-San Marco”, 95125 Catania, Italy; lore.aliotta@gmail.com (L.A.); gavazziliviomaria@gmail.com (L.M.G.); pietrofoti@hotmail.com (P.V.F.); spalmucci@unict.it (S.P.); mariachiaralg@gmail.com (M.C.L.G.); cor_spatola@hotmail.com (C.S.); corrado.ini@gmail.com (C.I.); tiralongofrancesco91@hotmail.it (F.T.); davidegiuseppecastiglione@gmail.com (D.C.); rbella@unict.it (R.B.); basile.antonello73@gmail.com (A.B.); 2 Department of Radiology, Azienda Ospedaliera Cannizzaro, 95126 Catania, Italy; piemalfa@alice.it; 3 Department of Diagnostic Imaging, Interventional Radiology and Neuroradiology, Azienda di Rilievo Nazionale Alta Specializzazione (ARNAS) Garibaldi, 95123 Catania, Italy; gianlucagalvano@gmail.com; 4 Department of Surgery and Medical-Surgical Specialties, University of Catania, 95123 Catania, Italy; giuseppe.lanza1@unict.it; 5 Department of Diagnostic Imaging, Sandro Pertini Hospital, 00157 Rome, Italy; adrenalina_1@hotmail.it; 6 Department of Radiological Sciences, Oncology and Pathology, Policlinico Umberto I, Sapienza University of Rome, 00161 Rome, Italy; vito.cantisani@uniroma1.it; 7 Department of Translational and Precision Medicine, Sapienza University of Rome, 00185 Rome, Italy

**Keywords:** carotid stenosis, plaque vulnerability, revascularization therapies

## Abstract

Carotid stenosis is a common pathology in clinical practice and unfortunately carries a high risk of serious cerebrovascular events. The early recognition of carotid plaque and, consequently, a careful analysis by means of multimodal imaging are the necessary steps to undertake a correct management pathway, aimed at preventing or, if not possible, reducing the risk of atherogenic phenomena responsible for cerebral infarction. In particular, the presence or absence of clinical symptoms, understood as the occurrence of events such as TIAs in the last 6 months, non-disabling strokes or repeated episodes of amaurosis fugax, and the degree of carotid stenosis, are certainly the most studied parameters, and as reported by several international guidelines, can lead to the best therapeutic strategy: whether to rely on conservative medical therapy or to resort to mechanical revascularization of the carotid stenosis. According to the recommendations of the European Society of Vascular Surgery, mechanical revascularization is recommended for stenosis > 50% in symptomatic patients and stenosis > 60% in asymptomatic patients. In contrast, the latest findings on plaque vulnerability have focused attention on individual patient characteristics and clinical comorbidities that may be responsible for plaque inflammation and should therefore be taken into consideration to decide if revascularization treatment is needed even in those subjects who present stenosis with less degree than reported as critical value. Moreover, further radiological investigations are fundamental to finding the presence of entities such as plaque ulceration, plaque neo-vascularization, fibrous caps, and intraplaque lipid core that are responsible for increased vulnerability. Medical therapy involves interventions aimed at eliminating cardiovascular risk factors by administering drugs that control the comorbidities responsible for worsening carotid stenosis. Recent studies are also evaluating the effectiveness of new plaque-modifying drugs or targeted anti-inflammatory agents in reducing the risk of plaque development and complications. Revascularization therapies, on the other hand, include surgery (CEA), the endovascular technique (CAS), and a new hybrid technique (TCAR): they are all valid alternatives for the treatment of carotid stenosis, each with specific technical difficulties, but on the whole with comparable safety profiles and risk rates of postoperative complications, although some recent emergencies have focused attention on possible short- and long-term gender-dependent outcome differences. The aim of this manuscript is to present the state of the art in the management of patients with carotid stenosis and to take a closer look at revascularization options. In our opinion, the choice of one strategy over another should therefore depend on gender, anatomical features of the patient, preoperative comorbidities, and last but not least, the experience of the center and the multidisciplinary team involved in the management of the patient.

## 1. Introduction

Carotid stenosis has become a common condition in daily medical practice due to both the aging of the general population and the increase in cardiovascular risk factors and is burdened with high morbidity and mortality as it can cause acute cerebrovascular events [1]. In fact, when the atherosclerotic plaque reaches 50% of the carotid lumen, it can induce a hemodynamically significant stenosis [2]; moreover, let us remember that atherosclerotic plaque is an inflammatory and metabolically active pathology capable of inducing thrombotic and/or embolic phenomena that can aggravate the stenosis and thus the cerebral blood flow, giving rise, we repeat, to cerebral ischemic phenomena [3].

According to data from the literature, although the elimination or correction of certain cardiovascular risk factors has helped to reduce the probability of ipsilateral cerebral infarction by about 1% per year, about 15% of them are nonetheless secondary to carotid stenosis [4], hence the importance of early diagnosis of the plaque to ensure the best available treatment and reduce the possibility of adverse ischemic events.

Nowadays, the availability of non-invasive imaging techniques has facilitated the diagnosis, allowing the detection of the plaque and a careful analysis of its characteristics and the degree of stenosis: the evaluation should be performed with ultrasound as a first-line examination, and in case of further investigation, computed tomography angiography (CTA) and/or magnetic resonance angiography (MRA) is recommended to assess the extent and severity of the extracranial carotid stenosis [2,5].

Based on the clinical and radiological data, it will be possible to decide on the most effective therapeutic management. The main options available are medical therapy, surgical therapy such as carotid endarterectomy (CEA), endovascular therapies such as carotid angioplasty and stenting (CAS), a new hybrid treatment option such as transcarotid artery revascularization (TCAR), or a combination of these. The possibility of performing minimally invasive vascular access, reducing operation and hospitalization time and healthcare costs, has led medicine to make greater use of endovascular therapies than traditional surgery: the indications for CEA and CAS are in fact very similar so that CAS represents a viable alternative to CEA in well-studied and selected subgroups of patients [6]. On the other hand, TCAR is a third viable treatment option for carotid plaque stenosis and has great interest due to the lower rates of perioperative complications compared to CEA, although, to date, a major limitation is the lack of proven experience between the various institutions.

The purpose of this paper is to analyze the treatment options for carotid stenosis, understand their indications and effectiveness, and understand how far less invasive treatments can be used.

## 2. An Overview of Carotid Stenosis Imaging

In the evaluation of carotid stenosis, the focus is on recognizing and distinguishing patients at low risk of stroke from those who appear to be at high risk; in fact, as suggested by the scientific literature, it seems that the former may benefit only from a conservative medical approach, while the latter may require operative management [7].

Among the risk factors for carotid plaque, the degree of stenosis is certainly the most studied parameter in clinical practice and can be calculated through various classification systems recognized in the literature:

- The North American Symptomatic Carotid Endarterectomy Trial (NASCET): A comparison of the stenotic segment with the normal distal diameter of the post-stenotic ICA [8];
- European Carotid Surgery Trial (ECST): A comparison of the diameter of the stenotic area with the normal diameter of the carotid bulb [9];
- Common carotid artery (CC): The measurement of the residual lumen diameter in the most stenotic portion of the artery and subsequent comparison with the lumen diameter of the proximal common carotid artery (CCA) [10].

However, new emerging parameters responsible for plaque vulnerability have recently been shown to influence plaque evolution, possibly toward worsening stenosis or acute occlusive phenomena [4].

The search for these parameters, such as intraplaque bleeding (IPH), ulceration, neovascularization, fibrous plug thickness, and the presence of a necrotic lipid core (LRNC), becomes mandatory as their presence may be an additional factor to the degree of stenosis in the decision toward operative management rather than simple medical therapy [11].

Consequently, future studies will focus on finding additional factors involved in plaque vulnerability and thus increased cerebrovascular risk, such as Wall Shear Stress (WSS) [12] or Plaque Loading, understood as the ability to divide the entire plaque into multiple modules and assess the volume of each subcomponent with different characteristics [2].

Carotid stenosis evaluation needs radiological investigations and can make use of multimodality imaging.

**Figure 1:**
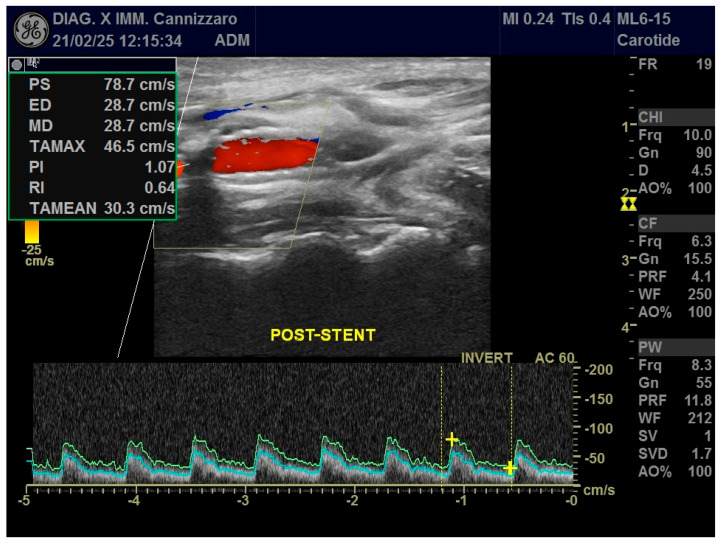
Ultrasound applications in a patient who has received CAS with stent positioning in the right ICA. Color Doppler and Power Doppler modes post-stent show significant hemodynamic stenosis resolution with normalization of VPS values and resistance indices.

Standard ultrasound, in its B-Mode and Color Doppler applications, is the first-line diagnostic investigation and can be used for both the initial assessment of pathology and follow-up after treatment (Figure 1). It is a widely available technique, free of ionizing radiation, which allows a rapid and dynamic assessment of both the morphologic features of the plaque and any related hemodynamic changes in blood flow, but it is highly dependent on operator experience. Another limitation of ultrasound is the inability to study a high-calcium plaque well [13].

Another application is contrast-enhanced ultrasound (CEUS), which, using the injection of a specific intravenous contrast agent (SONO-VUE, Bracco International B.V., Strawinskylaan 3051, NL-1077 ZX Amsterdam, Netherlands), allows us to obtain additional qualitative information such as the presence or absence of plaque irregularities or ulcerative phenomena, as well as assess and quantify intraplaque neovascularization, which is one of the vulnerability factors [14,15].

3D ultrasound and Vector Flow (VF) are the most advanced ultrasound techniques: the former, for example, can accurately assess plaque volume compared to conventional US, while the latter can calculate the shear wave power of the blood column at each point in the plaque wall by quantifying the WSS [2,12,16,17,18].

**Figure 2:**
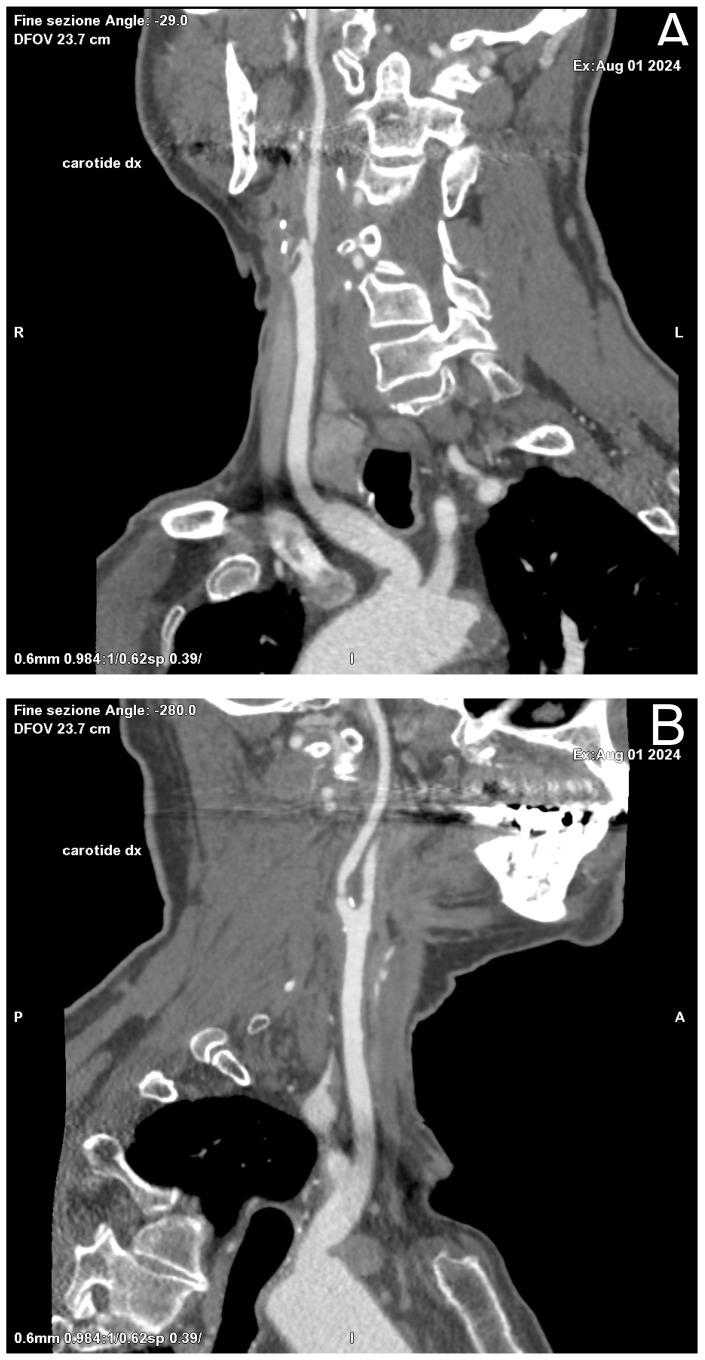
Oblique Coronal (**A**) and Sagittal (**B**) reformation performed for preoperative planning, which shows a mixed component plaque, determining stenosis of at least 50% in the right ICA in the post-ostial tract.

Among other diagnostic tests available for the evaluation of carotid stenosis, CT is often a second-choice test, performed as an adjunct to US. CT allows a more accurate and precise assessment of both plaque morphology and the degree of stenosis and, being relatively standardized across institutions and platforms, is not affected by operator influence. In addition, by providing a panoramic view of the entire vascular circle of the head and neck district, it allows us to analyze, in a single acquisition, both carotid arteries and the intracranial arterial circle, not accessible by ultrasound; moreover, it could be used for planning revascularization therapies thanks to reformatting software like MPR (Figure 2) and Volume Rendering (VR) (Carestream, ImageView Software, version 2.1, 150 Verona Street, Rochester, NY, USA).

The main disadvantages of CTA are the use of ionized radiation and an iodine contrast agent, potentially nephrotoxic and causing severe allergic reactions. Other negative aspects are the inability to perform hemodynamic studies of blood flow, due to the lack of temporal resolution, and the possibility of motion artifacts in uncooperative patients [19].

Recent developments in CT have provided software capable of calculating the plaque burden, which appears to be an emerging factor responsible for vulnerability [20].

Finally, MRI is a second-level investigation in the study of carotid plaque: the availability of multiple study sequences and the intravenous administration of a paramagnetic contrast agent, give us a multiparametric assessment of the plaque and better define the presence of vulnerability factors such as IPH, LNRH, fibrous caps, ulceration, and neovascularization. MRI has the advantage of being a safe examination as it does not use ionized radiation compared to CT, but instead requires longer acquisition times, with the possibility of motion artifacts, and is much less available and expensive [21,22]. However, new applications have introduced specific sequences that can study hemodynamic parameters such as WSS and may promote greater future use of MRI in the diagnostic-management pathway of carotid stenosis [23,24].

## 3. Indications and Treatments Available

Although the recent literature reports the experience of numerous clinical pharmacology and interventional trials, today the management of carotid stenosis is still debated; in fact, there is a certain variability of strategies in the prevention and management of strokes related to carotid stenosis between different medical specialties.

In clinical practice we have several therapies for the management of carotid stenosis: medical therapy, surgical therapy, endovascular therapy, T-CAR, or possibly a combination of the above [3]. Medical therapy is a conservative strategy aimed at eliminating cardiovascular risk factors, through lifestyle changes, and correcting any associated medical comorbidities, for which specific drugs are prescribed; in contrast, the other therapies mentioned above represent operative strategies for luminal arterial revascularization by eliminating or reducing the mechanical stenosis, obtained with different techniques and types of approach [4].

The first Carotid Revascularization Endartectomy vs. Stenting Trial (CREST), the Carotid Revascularization and Medical Management for Asymptomatic Carotid Stenosis Trial (CREST-2) [25], and the Stent Supported Percutaneous Angioplasty of the Carotid Artery Versus Endarterectomy 2 (SPACE 2) [26] represent the international clinical trials recently conducted to assess and compare best therapeutic management in patients with carotid stenosis. In particular, the SPACE-2 trial aimed to compare three groups of patients with carotid stenosis who had received, respectively, CEA or CAS plus medical therapy and only medical therapy, resulting in a very limited number of recruited patients for whom it has been terminated. The CREST study is a large randomized clinical trial that aims to compare the safety and efficacy of two different therapeutic strategies, such as CAS and CEA, in preventing ischemic events in patients with symptomatic and asymptomatic carotid stenosis, demonstrating nearly comparable results between the two groups of patients in terms of a combined primary endpoint of stroke, myocardial infarction, or death. The CREST-2 study was started in Canada and the USA in December 2014 and is structured as a double randomized clinical trial with double blinding aimed at defining the best therapeutic strategy in patients with significant stenosis (>70%) but who are asymptomatic; the first trial compared the group of patients who were undergoing CEA plus medical therapy with the group receiving only medical therapy, while the second trial compared the group of patients who received CAS plus medical therapy versus the one who received only medical therapy.

However, it seems reasonable that optimal patient care can be established through a multidisciplinary approach based on a careful and step-by-step analysis of all the factors related to plaque and potentially worsening blood flow in the brain [27].

Certainly, the first step is to identify the presence or absence of cardiovascular risk factors such as, for example, smoking and/or alcohol habits, a sedentary lifestyle, improper diet, and the presence of any medical comorbidities such as arterial hypertension, diabetes, dyslipidemia, connectivopathies, or fibromuscular dysplasia [28]. These factors seem to contribute actively to the evolution of carotid stenosis by activating inflammatory processes at the level of vessel wall, particularly the middle-intimate layer, and are able to precipitate in cerebral atherothrombotic phenomena: once identified, they must be removed or corrected [29,30,31].

The next step is to assess whether, in addition to conservative medical therapy, an operative type of intervention is necessary and, if so, to choose for each selected patient the most suitable approach and technique: whether surgical, endovascular, or T-CAR. In this sense, the current best practices recommended by different scientific institutions propose to categorize patients suffering from carotid stenosis according to whether they were symptomatic or not, where symptomatic refers to patients who had experienced cerebrovascular events such as non-disabling stroke, TIA in the last six months, or multiple episodes of amaurosis fugax [3].

In symptomatic patients, the latest indications suggest a revascularization for luminal stenosis > 50%, while in the case of asymptomatic patients, the target indicated is stenosis > 60% [4].

However, the introduction of the plaque vulnerability concept has highlighted that the degree of stenosis is often not a sufficient parameter in predicting the real risk of future cerebrovascular events, especially in asymptomatic patients; for this reason the presence or absence of entities such as IPH, neovascularization of plaque, ulceration, and fibrous caps may suggest the need for revascularization also for asymptomatic patients with stenosis < 60%, especially if between 50 and 59%. In this regard a study by Hackam et al. calls for the inclusion of this subtype of patients in revascularization trials so that more data can be extrapolated from them in the future [32].

On the other hand, some clinical trials such as the 2nd European Carotid Surgery Trial (ECST-2) [33] study have tried to assess whether the use of operative management such as CEA or CAS brings an effective benefit compared to only Optimized Medical Therapy (OMT) in patients with symptomatic carotid stenosis (>50%) by dividing patients into three risk classes, low, intermediate, and high, assigned through a risk score “CAR (Carotid Artery Risk)”. Although preliminary, these data have shown that patients with low or intermediate risk would not seem to benefit from revascularization therapies compared to the OMT alone [34].

## 4. Medical Therapy

The current state of the art in medical therapy for carotid stenosis involves eliminating or correcting cardiovascular risk factors and associated medical comorbidities, as already mentioned above. The IRIS (Insulin Resistance Intervention after Stroke) study showed that cessation of smoking causes a 44% reduction in the risk of stroke, myocardial infarction, or vascular death in 5 years [35]; in addition, according to the Lyon Diet Heart Study, a balanced “Mediterranean type diet” with no or minimal amounts of alcohol among people with coronary disease is able to reduce both stroke and recurrent myocardial infarction by >60% in 4 years [36].

For the control of hypertension and dysmetabolic diseases, such as diabetes and dyslipidemia, we have several drugs such as lipid-lowering interventions, antidiabetics, and antihypertensive medications.

Regarding LDL fat, for example, there is a target of 55 mg/dl to be achieved by using only statins, in the first line, and if necessary, adding another lipid-lowering drug such as ezetimibe, which has been approved in clinical trials [28,29].

Arterial hypertension is controlled using ACE inhibitors with a target systolic pressure < 130 mmHg in patients under 65 and diastolic pressure < 80 mmHg as secondary prevention [37].

Glycemic control in patients with carotid stenosis can be achieved using different classes of non-insulinemic hypoglycemic drugs. Among them, sodium-glucose cotransporter-2 inhibitors (SGLT-2is) and GLP-1 RA have been shown to reduce the risk of major cardiovascular adverse events (MACEs) and, therefore, are recommended at the forefront of both diabetes and international cardiovascular guidelines [38]. Thiazolidinediones, a class of insulin-sensitizing drugs, are MACE-neutral but may have benefits in preventing stroke. In contrast, dipeptidyl peptidase-4 inhibitor (DPP-4is) does not appear to have any beneficial effect on cardiovascular outcomes [39].

Another therapeutic option is antithrombotic drugs, although their use is still debated in asymptomatic patients. In this subgroup, Single AntiPlatelet Therapy (SAPT) with aspirin (Bayer AG. Kaiser-Wilhelm-Allee 1 51373 Leverkusen Germany) or clopidogrel appears to reduce atherothrombotic risk and has shown to be better than anticoagulants [40]. The ESC 2021 guidelines suggest long-term SAPT with low-dose aspirin in asymptomatic patients with stenosis >50% to reduce not only the risk of ipsilateral stroke but also other cardiovascular events whose risk is increased in the presence of carotid stenosis [41]. Dual AntiPlatelet Therapy (DAPT), obtained by a combination of aspirin and clopidogrel, showed no benefit compared to SAPT in this subgroup of asymptomatic patients [42]. In symptomatic subjects, who have already experienced acute cerebrovascular episodes such as minor strokes or TIA, DAPT seems to reduce the risk of recurrent stroke even if there is no evidence that it is safe in patients with underlying carotid stenosis [43]. DAPT can also be administered in patients with permanent carotid stents; in addition, a recent meta-analysis by Barkat M. et al., comparing DAPT to SAPT after carotid interventions, found a 13% reduction in the risk of TIA with DAPT compared to SAPT but no significant difference emerged in terms of stroke risk in the two subgroups. In contrast, there was an increased risk of bleeding and neck hematoma in patients who had received DAPT [44].

Finally, as inflammation is a promising target in atherothrombotic stroke prevention, new therapeutic strategies are focusing on the use of broad-spectrum and specific anti-inflammatory drugs, and on plaque-modifying drugs like micro-RNA (MiRNA). Among the broad-spectrum drugs, colchicine was the most studied in clinical trials with contradictory results and a non-negligible toxicity profile. Specific or targeted anti-inflammatory agents are monoclonal antibodies that target the main cytokines of interest in the regulation of the inflammatory process. MiRNAs act on gene transcription by regulating macrophage activation and inflammation, reducing plaque evolution. Although very promising in preclinical studies, these therapies have not yet shown the results expected in clinical trials and remain therefore still the subject of study for the future [45].

## 5. Revascularization Therapy: Surgical, Endovascular, or TCAR

According to the European Society of Vascular Surgery guidelines, the revascularization therapies for carotid stenosis should be performed within fourteen days after the “index” event; in particular, a meta-analysis by Coelho et al. shows a lower rate of stroke at thirty days when CEA and CAS were performed within the first two days after an index event rather than between the third and fourteenth day after the event [46].

Among operative therapies, CEA is certainly the most known technique in the scientific literature, reported for more than 60 years, and consists of the surgical removal of atherosclerotic material at the plaque level, which results in hemodynamically significant stenosis. CEA generally requires general anesthesia, with the resulting potential risks, although some highly experienced centers are able to perform CEA under local anesthesia by blocking the brachial plexus. The recent scientific literature has reported very encouraging data in this respect, suggesting comparable data between general and local anesthesia, even if there are some disadvantages such as the lack of remote access to the upper airway, the impossibility of converting regional anesthesia into general anesthesia when necessary, the need for patient collaboration, and the inadvertent subarachnoid or intravascular injection of local anesthetic [47].

There are currently two main methods for CEA packaging: conventional endarterectomy (C-CEA) and inverted endarterectomy (E-CEA). The conventional technique (C-CEA) is preferred, and it is performed through a longitudinal arteriotomy of the internal carotid artery (ICA) usually followed by a primary suture or, alternatively, by introducing a closure patch. In this regard, it appears that a primary suture increases the risk of restenosis at 5 years [48] while a recent meta-analysis by Rerkasem et al. has shown that a carotid patch angioplasty has reduced the combined perioperative and long-term risks of stroke and restenosis [49]. The reversal technique (E-CEA) is used as an alternative to the previous one and consists of a transverse arteriotomy and the anatomical reimplantation of the ICA in the carotid bulb following distal subversion and the removal of plaque. The potential advantage of this technique is that it does not need the placement of a closing patch.

Complications associated with CEA include the increased risk of myocardial infarction, embolic-related stroke, cranial nerve paralysis, permanent changes in sensitivity, infection, bleeding, and the possible formation of arterial pseudoaneurysms at the surgical suture [4]. In particular, the female gender would seem to be associated with a higher rate of a perioperative cerebrovascular accident (CVA)/stroke, although the sample should be homogenized, given the small number of women undergoing surgery compared to men [50].

Patients undergoing CEA require therapy with ATP and heparin during surgery, followed by ATP indefinitely.

Endovascular therapy is a valid alternative to surgery for the revascularization of carotid stenosis since it has the same indications as CEA and consists of an angioplasty and the stenting of the carotid plaque (CAS). Unlike CEA, it allows minimally invasive access and requires only local anesthesia, so it is often preferred in patients who do not have an optimal surgical and/or anesthesiologic profile; for example, patients with moderate to severe heart failure, respiratory failure, renal failure, high carotid bifurcation, previous dissection, irradiation of the neck, and hemodynamically significant control-lateral stenotic carotid disease [51].

CAS is relatively available and free from anesthetic risks, but it is not recommended in case of technical access difficulties such as the presence of a bovine arch, diffuse calcific atheromasia of the aortic arch, super thoracic trunks, or vascular tortuosity. It is also contraindicated in the case of comorbidities such as severe renal failure (EGR < 30 mL/min), a pre-existing aneurysm or vascular dissection, and a proven severe allergy to an iodized contrast medium. Modern studies have focused on plaque vulnerability as a possible high-risk factor for CAS [52].

**Figure 3:**
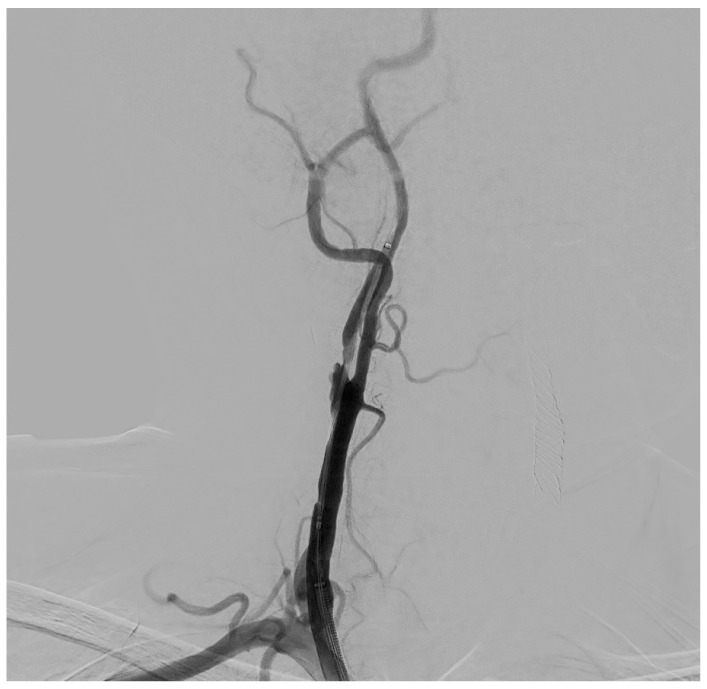
Selective DSA (Digital Subtraction Angiography) of right CCA showing significative (>50%) stenosis of right ICA, in post-ostial tract.

**Figure 4:**
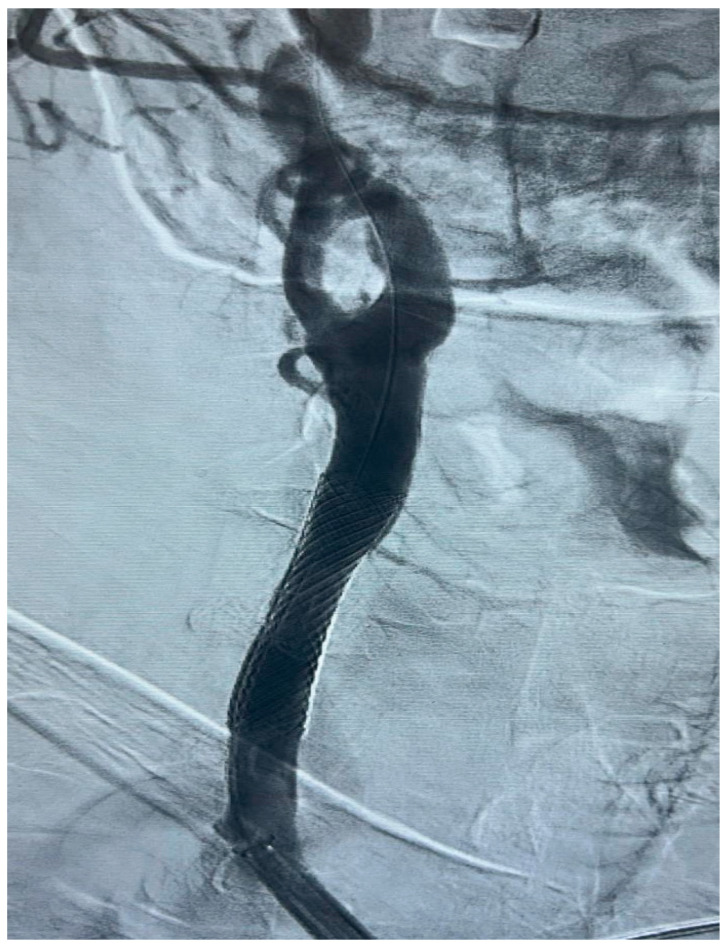
DSA showing a coaxial umbrella-like filter (Boston Scientific Filterwire Ez, 300 Boston Scientific Way Marlborough, MA 01752-1234), positioned in the distal ICA, to prevent embolic phenomena during the balloon angioplasty and stent positioning of the carotid stenotic plaque.

**Figure 5:**
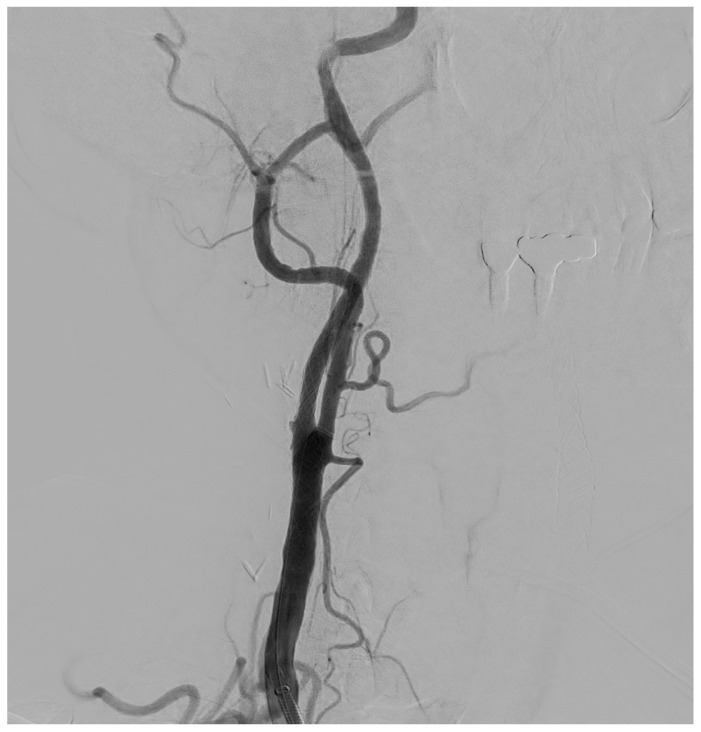
Final DSA control, in a patient with significant (>50%) stenosis of the ICA who has been treated by positioning a closed-cell stent (Boston Scientific Wallstent, 8 × 30 mm, 300 Boston Scientific Way Marlborough, MA 01752-1234) dilated with a balloon catheter (Ultraverse PTA dilatation catheter 6 × 20 mm, Becton, Dickinson and Company’s, 1 Becton Drive, Franklin Lakes, New Jersey 07417, USA).

The procedure is performed in the angiographic suite, under mild sedation to monitor the neurological status of the patient, by arterial vascular access, usually femoral. With the wire–catheter system, the common carotid artery (CCA) of the pathological side is selectively catheterized and a diagnostic angiography is performed to identify the stenosed area (Figure 3). The stenosis carotid tract is crossed through the wire–catheter system, and only after the placement of a neuroprotection device in the ICA, a balloon angioplasty is performed to compress the atheromatic lesion and restore the original luminal vessel diameter. Regarding neuroprotection, today we have two classes of embolic protection devices available: a coaxial umbrella-like filter positioned in the distal ICA (Figure 4) or hemodynamic balloon block systems with reversal flow that can be placed either proximally or distally to the carotid stenosis with overlapping results in reducing the distal embolic phenomena risk but with a higher rate of periprocedural vasospasm in the case of a distal umbrella-like filter [53]. At this point, it is necessary to inject 1 mg of atropine to avoid vasospasm before releasing a permanent endovascular stent. We have three types of endovascular stents such as open-cell, closed-cell, or double-layer (Figure 5). Open-cell stents were worse than closed-cell due to the higher degree of plaque protrusion and the procoagulant state induced by the stent that can trigger embolic phenomena, recording a higher rate of stroke or restenosis at 30 days. The double-layer stents are still in the process of approval by the FDA although they have shown in some clinical trials strongly encouraging results with a risk of adverse events at 30 days <2% [54].

Generally, patients undergoing CAS receive both DAPT and heparin therapy during the procedure, with DAPT continuing for about 3 months after intervention and subsequent SAPT indefinitely.

Regarding periprocedural complications, stroke is certainly the most feared, especially in the first 28–30 days after treatment; less frequently it is possible to find acute myocardial infarction, bradycardia and hypotension, arterial dissection, bleeding, hematoma, or an arterial pseudoaneurysm at the puncture site [55]. Postoperative CVA/stroke has shown a higher risk rate than in CEA; moreover, unlike the latter in CAS, there does not appear to be gender differences or female predilection [50].

In 2015, the Food and Drug Administration (FDA) approved transcarotid artery revascularization (TCAR) as a third option in the operative management of extracranial carotid artery stenosis; however, data suggest that in centers capable of performing all three revascularization procedures, one in four patients undergoes TCAR [56].

TCAR is a treatment option that is less invasive than CEA and can be performed under general anesthesia or more simply with local anesthesia. The procedure begins with a small incision just above the collarbone and placing an 8Fr vascular introducer directly in the carotid artery, in an area far from disease. Then, the vascular sheet is connected to a transcarotid neuroprotection system (ENROUTE™ Transcarotid Neuroprotection System, Boston Scientific, 300 Boston Scientific Way Marlborough, MA 01752-1234), placed outside the patient, and through the same connection, is connected to an additional vascular introducer previously placed in the femoral vein. This external circuit allows blood flow to be filtered and reversed away from the brain, so any atherothrombotic debris is pushed and trapped in the filter of the neuroprotective system before returning to the femoral vein, reducing the stroke risk.

Finally, a stent is inserted to open the stenotic carotid artery and restore the luminal diameter [57].

In the postoperative period, patients require intensive hemodynamic monitoring and periodic neurological evaluation, using vasodilators if necessary to reduce blood pressure and avoid cerebral hyper-perfusion, or anticholinergic drugs in case of bradycardia, secondary to the dysregulation of baroreceptors induced during manipulation.

The main contraindications to TCAR are severe carotid artery calcification or vessel tortuosity, significant thrombus within the lesion, metal allergies to the device, the presence of tracheostomy, or the violation of anatomical criteria. Anatomical criteria comprise the need to have a diameter of the carotid artery between at least 4 and 9 mm, a resizing of the proximal vessel tract to at least 12 mm, to prevent stent migration, and a working distance between the site of incision and the upper margin of the clavicle of at least 5 cm. In this regard, the correct selection and identification of patients who are candidates for TCAR is fundamental.

The advantages of TCAR compared to CEA are minimally invasive access with a small surgical scar, shorter time operators, and lower risk of injury to the cranial nerves. Regarding complications, several multicenter studies found that patients undergoing TCAR revascularization showed a lower risk of perioperative stroke (1.3–2.0%), mortality (0.48–0.7%), and myocardial infarction (0.4–0.57%) than CEA [58,59,60]. In general, TCAR should be preferred in patients with complex vascular anatomy such as aortic arch type II, or even more so, type III, since in these cases CAS may be difficult to perform technically [60].

Finally, the TCAR technique requires antithrombotic therapy, in particular, the SVS guidelines provide for DAPT within 5 days before the procedure, using a combination of aspirin and P2Y12 inhibitors, DAPT for 1 month after the intervention, and then aspirin indefinitely [61,62].

Below we show a summary table (Table 1) of the main indications for the operative treatment of carotid stenosis with emphasis on the main advantages and disadvantages related to the different techniques mentioned above [63,64,65].

## 6. Discussion

In everyday clinical practice, patients with carotid stenosis deserve careful management aimed to reduce the likelihood of related acute cerebrovascular events, often fatal or severely disabling.

Plaque evaluation should be multifactorial and consider any characteristic that may modify its pathophysiology and consequently its hemodynamics. However, there is variability in the strategies for preventing and managing stroke related to carotid stenosis among medical specialties. Optimal patient care can only be achieved by establishing standardization through guidelines shared by different specialties such as neurology, stroke medicine, cardiology, angiology, ophthalmology, vascular surgery, interventional radiology, and neurosurgery. The search for a personalized cardiovascular risk profile, taking into account individual characteristics, the presence of associated comorbidities, and other factors responsible for plaque inflammation and vulnerability, should be considered to classify patients within the corresponding risk class. In this sense, the ECST-2 clinical trial, although underway but with encouraging results, is proposed to chart the way forward, although it has several limitations due to the fact that among the risk factors considered by CAR, only plaque ulceration is included, excluding others like IPH, fibrous caps, and neovascularization, and which may, on the other hand, suggest a higher cardiovascular risk than ECTS-2 [34].

Another very important aspect is patient education to know and avoid the risk factors determining plaque worsening so they could be actively involved in the decision process for the implementation of the best therapeutic options.

Patients must be educated to change their lifestyle and diet, to periodically check blood pressure, glycemic and lipidemic values, through appropriate laboratory analysis, and if necessary, to properly take the pharmacological therapy indicated for the management of possible medical comorbidities such as hypertension, diabetes, or dyslipidemia.

The need for operative revascularization therapy should be carefully studied, considering the possible advantages and disadvantages of the three different therapeutic options available; in this sense, the CREST-2 [25] study appears to be an excellent starting point for establishing the best future guidelines on the management of these patients, especially to select patients who could benefit by OMT, but needs further implementation and major recruitment of patients. In patients with an indication for revascularization, the type of technique should be chosen considering the patient’s age, gender, present comorbidities, surgical anesthetic and hemorrhagic risk, the presence or absence of vascular anatomical variants, and the risks associated with each of the three available therapeutic options.

Although CEA and CAS showed equivalent results in the CREST study [43], it appears that CEA is associated with a higher risk of AMI due to surgical stress on the heart, while CAS is associated with a higher rate of perioperative stroke especially in subjects aged > 70 years. It seems obvious that CAS should be preferred in subjects with a high cardiac risk profile while CEA should be preferred in subjects aged over 70 years.

Indeed, a 2018 meta-analysis conducted by Meershoek et al. in the MEDLINE, EMBASE, and Cochrane Library databases showed that within 30 days of treatment, strokes or deaths were reported in 1.8% in the group undergoing carotid endarterectomy (CEA) and 2.2% in the group undergoing carotid artery stenting (CAS) [66].

An updated systematic review and meta-analysis in 2023 by Vasavada AM et al. confirmed that endarterectomy had fewer harmful effects than stenting, although stenting had better outcomes in terms of myocardial infarctions [67].

Also in 2023, a major meta-analysis conducted by Li W et al. on seventeen randomized controlled trials with 12,277 participants (6514 and 5763 in the CAS and CEA groups, respectively) demonstrated that compared with CEA, CAS was associated with decreased risks of perioperative MI (RR = 0.47, 95% CI = 0.29∼0.77) and perioperative cranial nerve palsy (RR = 0.02, 95% CI = 0.01∼0.06) but higher risks of perioperative stroke (RR = 1.48, 95% CI = 1.18∼1.87) and cumulative incidence of death or stroke (RR = 1.52, 95% CI = 1.20∼1.93). They concluded that perioperative safety was equivalent between CAS and CEA; however, CEA may be preferred when considering both procedural safety and long-term efficacy in preventing recurrent stroke [68].

Moreover, the female gender may be associated with a higher risk of postoperative CVA/stroke in patients undergoing CEA, suggesting the possibility of preferring CAS in women since they have not found differences in the risk rates related to gender [50], but a greater inclusion of female patients is needed to confirm the preliminary data. However, in our opinion, supported by some studies in the literature, CAS should be performed in high-volume centers, where more than 50 procedures a year are performed, since the rate of complications is related to the experience of the operator.

Finally, TCAR represents a third treatment option for carotid stenosis revascularization, allows minimally invasive access, and can be considered valid for high-risk surgical subjects.

Considering the wide variability of anatomical and physiopathological factors that come into play in the management process of the patient suffering from carotid stenosis, we think that in order to ensure the best revascularization treatment, it is necessary to refer patients with a high-risk profile to centers that have a multidisciplinary team of experts in all three therapeutic options.

## Figures and Tables

**Table 1:** Summary table of indications, advantages and disadvantages of the main revascularization carotid therapies.

Procedures	Main Indication	Advantage	Disadvantage
**CEA**	Symptomatic stenosis ≥ 50%, or asymptomatic ≥ 70% in patients with acceptable surgical risk.	Gold standard for patients with acceptable surgical risk. Proven long-term stroke risk reduction (5-year benefit in both symptomatic and selected asymptomatic patients) Lower perioperative stroke risk (1–3% in asymptomatic, 3–5% in symptomatic patients).	Invasive: requires cervical incision and arterial clamping. Risk of cranial nerve injury (2–3%). Longer recovery time and hospital stay compared to endovascular approaches.
**CAS**	Symptomatic ≥ 50% or asymptomatic ≥ 70% in patients unsuitable for CEA due to anatomy or comorbidities.	Minimally invasive; avoids cervical incision and general anesthesia. Appropriate for high-surgical-risk patients (e.g., severe comorbidities, prior neck surgery).	Higher perioperative stroke/death rate (~65% greater than CEA). Particularly elevated stroke risk in patients > 70 years. Risk of embolization due to arch manipulation and filter crossing. Long-term restenosis risk; requires duplex surveillance.
**T-CAR**	Symptomatic 70–99% in high surgical risk (age > 80, prior neck surgery/radiation). Also viable for complex anatomies in asymptomatic patients.	Minimally invasive: small incision at the base of the neck. Direct carotid access avoids aortic arch manipulation. Flow reversal provides neuroprotection prior to lesion crossing; fewer DWI-detected embolic lesions than CAS. Comparable 30-day stroke/death rate to CEA in the elderly (e.g., 1.5% in patients ≥ 80 years). * Significantly lower cranial nerve injury rate (≈0.3–0.6%). ** Shorter operative time and hospital stay (often ≤ 1 day). *** Cost-effective over 5–6 years in selected populations.	Not suitable in all anatomies (e.g., short or diseased common carotid segments). Challenging in “hostile necks” (e.g., prior radiation, scar tissue). Long-term outcome data still maturing; some studies suggest slightly higher 1-year stroke/death compared to CEA. Higher upfront cost than CEA.

* Dakour-Aridi, H.; Kashyap, V.S.; Wang, G.J.; Eldrup-Jorgensen, J.; Schermerhorn, M.L.; Malas, M.B. The impact of age on in-hospital outcomes after transcarotid artery revascularization, transfemoral carotid artery stenting, and carotid endarterectomy. *J. Vasc. Surg.* **2020**, *72*, 931–942.e2 [63], ** Wu, H.; Wang, Z.; Li, M.; Sun, P.; Wei, S.; Xie, B.; Zhang, C.; Zhang, L.; Bai, H. Outcomes of transcarotid artery revascularization: A systematic review. *Interv. Neuroradiol.* **2024**, *30*, 396–403 [64], *** Mehta, A.; Patel, P.B.; Bajakian, D.; Schutzer, R.; Morrissey, N.; Malas, M.; Schermerhorn, M.; Patel, V.I. Transcarotid artery revascularization versus carotid endarterectomy and transfemoral stenting in octogenarians. *J. Vasc. Surg.* **2021**, *74*, 1602–1608 [65].
